# Characterization of Luteinizing Hormone and Luteinizing Hormone Receptor and Their Indispensable Role in the Ovulatory Process of the Medaka

**DOI:** 10.1371/journal.pone.0054482

**Published:** 2013-01-23

**Authors:** Katsueki Ogiwara, Chika Fujimori, Sanath Rajapakse, Takayuki Takahashi

**Affiliations:** 1 Laboratory of Reproductive and Developmental Biology, Faculty of Science, Hokkaido University, Sapporo, Japan; 2 Department of Molecular Biology and Biotechnology, Faculty of Science, University of Peradeniya, Peradeniya, Sri Lanka; Glasgow Caledonian University, United Kingdom

## Abstract

The molecular properties and roles of luteinizing hormone (Lh) and its receptor (Lhcgrbb) have not been studied for the medaka (*Oryzias latipes*), which is an excellent animal model for ovulation studies. Here, we characterized the medaka Lh/Lhcgrbb system, with attention to its involvement in the ovulatory process of this teleost fish. In the medaka ovary, follicle-stimulating hormone receptor mRNA was expressed in small and medium-sized follicles, while *lhcgrbb* mRNA was expressed in the follicle layers of all growing follicles. Experiments using HEK 293T cells expressing medaka Lhcgrbb *in vitro* revealed that gonadotropin from pregnant mare’s serum and medaka recombinant Lh (rLh) bound to the fish Lhcgrbb. The fish gonadotropin subunits Gtha, Fshb, and Lhb were essentially expressed at fairly constant levels in the pituitary of the fish during a 24-h spawning cycle. Using medaka rLh, we developed a follicle culture system that allowed us to follow the whole process of oocyte maturation and ovulation *in vitro*. This follicle culture method enabled us to determine that the Lh surge for the preovulatory follicle occurred *in vivo* between 19 and 15 h before ovulation. The present study also showed that oocyte maturation and ovulation were delayed several hours *in vitro* compared with *in vivo*. Treatment of large follicles with medaka rLh *in vitro* significantly increased the expression of Mmp15, which was previously demonstrated to be crucial for ovulation in the fish. These findings demonstrate that Lh/Lhcgrbb is critically involved in the induction of oocyte maturation and ovulation.

## Introduction

Luteinizing hormone (LH) and follicle-stimulating hormone (FSH) are glycoprotein hormones, called gonadotropins, that regulate gonadal functions [1]. They are heterodimeric hormones composed of a common α-subunit that is non-covalently associated with a hormone-specific β-subunit [2]–[5]. Because of their importance in the regulation of reproduction, including ovulation, they have been studied in many species. cDNA sequences for gonadotropin subunits have been determined in 56 teleost fish species representing at least 14 teleost orders [1]. Gonadotropins exert their action through gonadotropin receptors, the LH receptor (LHCGRBB) and the FSH receptor (FSHRA). The receptors belong to the G protein-coupled cell surface receptor superfamily with seven transmembrane domains. In many species, LHCGRBB and FSHRA are primarily expressed in reproductive organs and act coordinately to control steroidogenesis, folliculogenesis, and ovulation. It is generally believed that in teleosts, Fshra is expressed in the granulosa cells of the ovary, whereas Lhcgrbb is expressed primarily in the theca and granulosa cells of preovulatory ovarian follicles [6]–[8]. The Gs/cAMP/PKA pathway is the basic signaling pathway of gonadotropin receptors. However, various novel pathways in gonadotropin receptor signaling have been reported recently [9].

Medaka (*Oryzias latipes*), which is a small egg-laying freshwater teleost, is a good model system for various fields of biology [10]–[12]. Recently, a draft of the medaka genome sequence has been determined [13] and is available. Furthermore, the fish has advantages for studying unsolved problems in reproductive biology, particularly in ovulation: (i) It spawns every day under suitable light and temperature conditions. (ii) The timing of the sequential processes of spawning, such as completion of vitellogenesis, breakdown of the germinal vesicle and ovulation, can be determined. (iii) Experimental systems for studying ovulation with whole ovaries and dissected follicles *in vitro* are available [14]–[16]. (iv) The proteolytic enzymes responsible for follicle rupture in ovulation have been determined [15]. It is productive to use a model animal to explore the molecular mechanisms and endocrine regulation of ovulation.

Ovulation, an important biological event in the ovary, is defined as the discharge of a mature oocyte from the ovarian follicle into the ovarian cavity or into the abdominal cavity, depending on the species [17], [18]. This process, which is triggered by LH, is achieved through a series of signaling pathways [17]. LH controls the expression of a variety of genes essential for ovulation, such as genes that code for steroidogenic enzymes [19], [20] and many other factors, including vascular endothelial growth factor [21], [22]. Matrix metalloproteinases (MMPs) and their inhibitors, tissue inhibitors of metalloproteinases (TIMPs), which are implicated in follicle rupture during ovulation, are also thought to be regulated by LH [23], [24].

We were interested in the endocrine mechanism underlying follicle rupture during ovulation in the medaka. However, there have been no studies of the gonadotropins or their receptors in the medaka. We therefore initiated the current study to characterize the fish gonadotropins and their receptors. Further, we examined the effects of gonadotropins on the expression of MMPs and TIMP-2b, which are critical in fish ovulation [15], using an *in vitro* follicle culture system recently developed for the medaka using recombinant medaka luteinizing hormone (the official symbols are *lh* for the gene and Lh for the protein). Our data indicate that among the MMPs examined and TIMP-2b, only MT2-MMP (the official symbols are *mmp15* for the gene and Mmp15 for the protein) is upregulated by Lh in the preovulatory follicles that are destined to ovulate.

## Results

### Three gonadotropin subunits and two gonadotropin receptors of medaka

While the medaka *gtha*, *fshb*, and *lhb* cDNAs isolated in the present study had nucleotide sequences corresponding to those available from the NCBI database, there were one and nine nucleotide substitutions for *fshra* and *lhcgrbb*, respectively. For *fshra*, at position 2020, the A (NCBI database) was changed to G (our sequence), resulting in the replacement of Tyr (NCBI) by Cys (our sequence) at position 671. The nine nucleotide substitutions for the *lhcgrbb* sequence were as follows: G (NCBI) to A (current study) at position 151, C to A at 297, C to T at 785, G to A at 936, A to G at 1323, T to C at 1629, C to G at 1695, T to C at 1837, and C to T at 1895. As a result, amino acid residue replacements occurred at the following three positions: Gly (NCBI) to Asp (current study) at position 50, Gly to Ser at 321, and Ser to Phe at 631. The open reading frames of the clones encoded proteins of 137 (Gtha), 117 (Fshb), 146 (Lhb), 687 (Fshra) and 688 (Lhcgrbb) amino acid residues. These putative proteins shared common domain structures with other vertebrate species (data not shown). The amino acid sequences of the medaka proteins were 42–50% (Gtha), 22–35% (Fshb), 32–47% (Lhb), 47–55% (Fshra), and 47–49% (Lhcgrbb) identical to those from other vertebrate species (Table 1).

**Table 1:** Amino acid sequence identity (%) between the medaka gonadotropin subunit and gonadotropin receptor proteins and those of other vertebrate species.

Protein	Human	Mouse	Chicken	*Xenopus*	Zebrafish
Gtha	43	47	45	42	50
Fshb	23	24	27	22	35
Lhb	36	32	32	43	47
Fshra	49	47	49	49	55
Lhcgrbb	48	48	49	47	47

Human (NP_001239312), mouse (NP_034019), chicken (XP_429886), *Xenopus* (NP_001085173), and zebrafish (NP_991250) for Gtha; human (NP_000501), mouse (NP_032071), chicken (NP_989588), *Xenopus* (NP_001084494), and zebrafish (NP_991187) for Fshb; human (NP_000885), mouse (NP_032523), chicken (ADY03193), *Xenopus* (NP_001079224), and zebrafish (AAV31153) for Lhb; human (NP_000136), mouse (NP_038551), chicken (NP_990410), *Xenopus* (NP_001243189), and zebrafish (AAP33512) for Fshra; and human (AAA59515), mouse (EDL38652), chicken (NP_990267), *Xenopus* (NP_001243190), and zebrafish (AAI62452) for Lhcgrbb.

### Expression of gonadotropin subunits in the tissues of medaka

**Figure 1:**
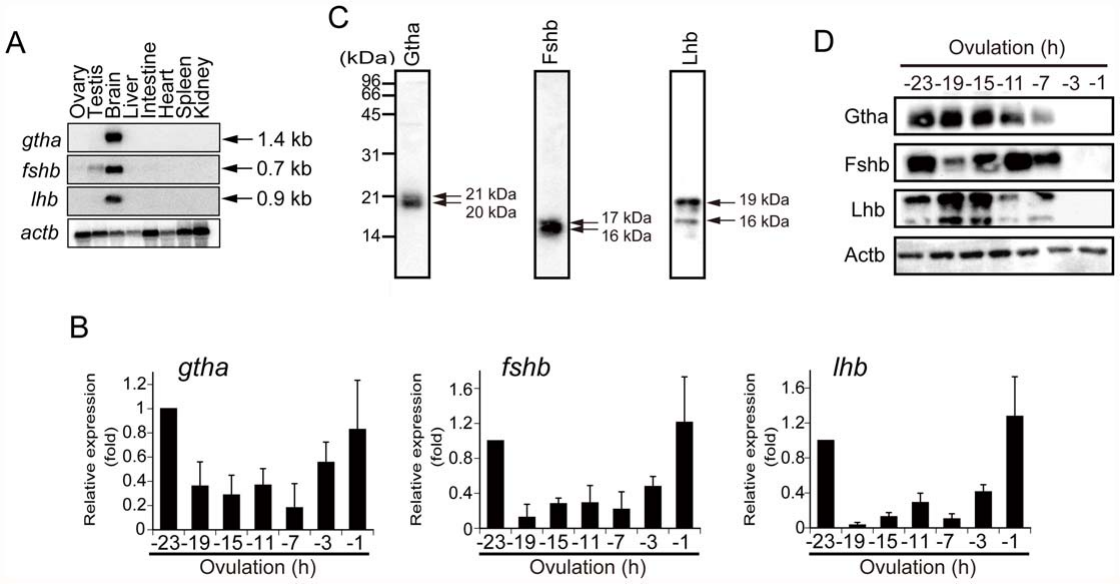
Expression of gonadotropins in the medaka pituitary. (**A**) Northern blot analyses of *gtha*, *fshb*, and *lhb* mRNAs were conducted using total RNAs isolated from various medaka tissues. Transcripts of *actb* were also detected as a control. The RNA was loaded at 60 µg/lane. (**B**) Real-time RT-PCR for *gtha*, *fshb*, and *lhb* were conducted using total RNAs (2.5 µg each) isolated from pituitaries of spawning medaka. The expression levels were normalized to that of *actb* and expressed as the fold change from the levels of the −23 h pituitary. The results are presented as the mean ±S.E.M. (n = 5). (**C**) Western blot analyses of gonadotropin subunit polypeptides were conducted using extracts prepared from the pituitaries of spawning medaka 1 h after ovulation. The proteins were loaded at 5 µg/lane. The polypeptides detected by specific antibodies for Gtha (left panel), Fshb (center panel), and Lhb (right panel) are shown. The sizes of the polypeptides are indicated at the right and those of the standard marker proteins at the left. (**D**) Western blot analyses of gonadotropin subunits were conducted using pituitary extracts isolated from spawning medaka at the indicated time points. The proteins were loaded at 5 µg/lane. Polypeptides were detected with specific antibodies. Medaka Actb was also detected as a control. The reproducibility of the findings was confirmed by conducting four separate experiments. The results of a representative experiment are presented.

Northern blot analysis for *gtha*, *fshb*, and *lhb* mRNA expression was conducted using total RNAs isolated from various medaka tissues. Among the tissues examined, the brain expressed transcripts of all of the gonadotropin subunits (Fig. 1A). Transcripts of *fshb* were slightly expressed in the testis. To determine their expression levels during a 24-h spawning cycle, real-time RT-PCR analysis was conducted using total RNAs isolated from fish pituitaries at the indicated time points. The expression levels of all of the subunit transcripts in the pituitary were high around the time of ovulation, whereas they were low in the time between 19 and 3 h before ovulation (Fig. 1B). Western blot analysis was performed on the pituitary extracts using specific antibodies raised against the respective subunits. Two polypeptides of about equal size (20 and 21 kDa for Gtha, 16 and 17 kDa for Fshb, and 16 and 19 kDa for Lhb) were visualized at the positions expected for each subunit (Fig. 1C). All the gonadotropin subunits were detected at fairly constant levels in the pituitary during a 24-h spawning cycle, except that the proteins were not detected in the tissue extract 3 and 1 h before ovulation (Fig. 1D). None of the antibodies detected any polypeptides when the ovary and testis extracts were used for the analysis, suggesting that the subunit protein levels in the fish gonads, if any, were too low to detect (data not shown).

### Expression and localization of gonadotropin receptor transcripts and proteins in the ovary

**Figure 2:**
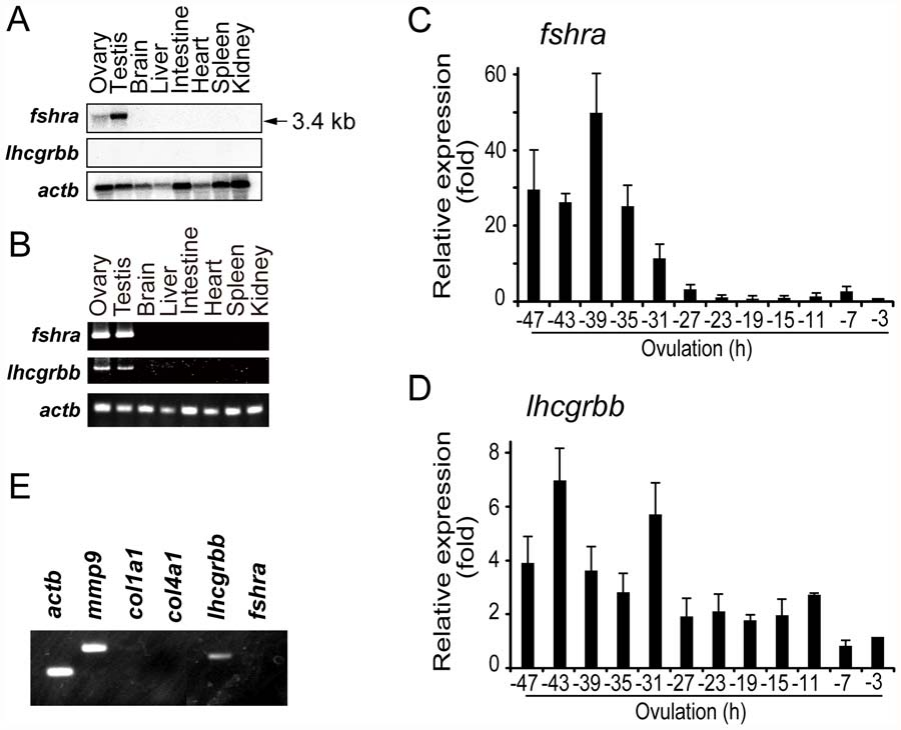
Expression of gonadotropin receptors in the medaka. (**A**) Northern blot analyses for *fshra* and *lhcgrbb* mRNAs were performed using total RNAs isolated from various medaka tissues. Transcripts of *actb* were also detected as a control. The RNAs were loaded at 60 µg/lane. The reproducibility of the findings was confirmed by conducting two separate experiments, and the results of one experiment are presented. (**B**) RT-PCR analysis was conducted for *fshra* and *lhcgrbb* mRNAs using total RNAs (2.5 µg each) isolated from various tissues of the medaka. For PCR of *fshra* and *lhcgrbb*, 30 cycles were used, and for *actb*, 20 cycles were used. The reproducibility of the findings was confirmed by conducting four separate experiments, and the results of a representative experiment are presented. (**C**) Real-time RT-PCR for *fshra* was conducted using total RNAs (2.5 µg each) isolated from follicles of spawning medaka ovaries. Ovarian follicles (stage VII to X) were collected every 4 h from the ovaries. The expression levels were normalized to that of *actb* and expressed as the fold change from the levels of the −3 h follicle. The results are presented as the mean ±S.E.M. (n = 5). (**D**) Real-time RT-PCR for *lhcgrbb* was conducted as in (**C**). The results are presented as the mean ±S.E.M. (n = 5). (**E**) RT-PCR analysis of *lhcgrbb* and *fshra* was conducted using total RNA (2.5 µg) isolated from the granulosa cells of large ovarian follicles. For comparison, the expression of *mmp9*, *col1a1*, *col4a1*, and *actb* (as a control) was also examined. The reproducibility of the findings was confirmed by conducting four separate experiments, and the results of a representative experiment are presented.

The temporal and spatial expression of gonadotropin receptor mRNA in the fish ovary was examined. Northern blot analysis using total RNAs isolated from various tissues of the fish revealed that a 3.4-kb transcript for *fshra* was abundantly expressed in the testis (Fig. 2A). The ovary also expressed *fshra* mRNA, although its level was low. In contrast, no signal for *lhcgrbb* mRNA was detected in any of the tissues examined. However, the expression of *lhcgrbb* mRNA in the ovary was demonstrated by RT-PCR (Fig. 2B). Changes in *fshra* and *lhcgrbb* mRNA levels were assayed by real-time RT-PCR using follicles collected from spawning fish ovaries every 4 h during the last 48 h before ovulation. The level of *fshra* transcript was relatively high between 47 (vitellogenic follicles at stage 8) and 31 h (postvitellogenic follicles at stage 9), but *fshra* mRNA expression rapidly decreased thereafter (Fig. 2C). In contrast, the *lhcgrbb* mRNA level as a whole tended to decrease during the 48 h period but was detectable until the time of ovulation (Fig. 2D). To gain insight into the localization of the *fshra* and *lhcgrbb* transcripts in the large preovulatory follicles of the fish, an RT-PCR analysis was conducted using cDNA prepared from the primary granulosa cells. A PCR product specific for *lhcgrbb* mRNA was amplified, whereas no product was detected for *fshra* mRNA (Fig. 2E). We also analyzed the transcripts of gelatinase B (*mmp9*), a marker for granulosa cells of the large follicles [25], and collagen type I α1-chain and collagen type IV α1-chain, markers for theca cells [26], [27]. A fragment of *mmp9*, but not of collagen type I α1-chain or collagen type IV α1-chain, was amplified, confirming that *lhcgrbb* mRNA, but not *fshra* mRNA, is expressed in the granulosa cells of the large follicles.

**Figure 3:**
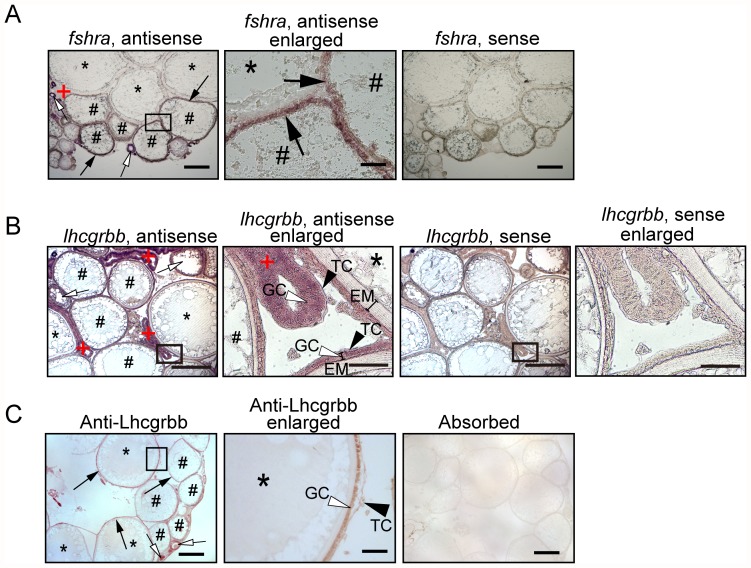
Histological localization analysis of gonadotropin receptors in the medaka ovary. (**A**) *In situ* hybridization analysis for *fshra* mRNA was conducted using frozen sections of the ovary isolated 1 h after ovulation. Staining with the antisense probe is shown in the left and middle panels. The boxed area in the left panel is shown at higher magnification in the middle panel. As a control, staining with the sense probe is shown in the right panel. Black arrows indicate positive staining associated with the follicle layers of middle-sized follicles, and white arrows indicate positive staining associated with the cytoplasm of small follicles. Bars represent 500 µm (for the right and left panels) and 100 µm (for the middle panel). *, large preovulatory follicle; #, medium-sized follicle; +, postovulatory follicle with no oocyte. (**B**) *In situ* hybridization analysis for *lhcgrbb* mRNA was conducted using paraffin sections of the ovary isolated 1 h after ovulation. The left two panels and right two panels show the staining with the antisense and sense probe, respectively. The boxed areas in the antisense and sense staining are also shown at higher magnification to the right. The white arrows indicate positive staining associated with the cytoplasm of small follicles. The black arrowhead and the white arrowhead in the enlarged antisense staining panel indicate the theca cell layer (TC) and granulosa cell layer (GC), respectively, that surround the egg membrane (EM) of follicles. Bars represent 400 µm (for the low-magnification panels) and 50 µm (for the higher-magnification panels). *, large preovulatory follicle; #, middle-sized follicle; +, postovulatory follicle with no oocyte. (**C**) Immunohistochemical analysis for Lhcgrbb was conducted using paraffin sections of the ovary isolated 1 h after ovulation. Staining with the antibody for medaka Lhcgrbb is shown in the left and middle panels. The boxed area in the left panel is shown at higher magnification in the middle panel. As a control, staining with the absorbed antibody is shown in the right panel. Black arrows indicate positive staining associated with the follicle layers of large and medium-sized follicles, and white arrows indicate positive staining associated with the cytoplasm of small follicles. The black arrowhead and the white arrowhead indicate the TC and GC layers, respectively. Bars represent 500 µm (for the right and left panels) and 100 µm (for the middle panel). *, large preovulatory follicle; #, middle-sized follicle; +, postovulatory follicle with no oocyte. The reproducibility of all findings was confirmed by conducting four separate experiments. The results of a representative experiment are presented.

By *in situ* hybridization analysis, *fshra* was localized in the follicle layer of small and medium follicles, but no signal was detected in the large follicles (Fig. 3A, left and middle panels). Using the sense probe for *fshra* mRNA, no signal was detected (Fig. 3A, right panel), indicating that the staining with the antisense probes used was specific. Ovarian expression of *lhcgrbb* mRNA was examined by *in situ* hybridization analysis. Positive signals were found in association with the follicle layer of all sizes of growing follicles when the staining with the sense and antisense probes was carefully compared (Fig. 3B, left two panels vs. right two panels). The signals were also detected in the oocyte cytoplasm of follicles smaller than 150 µm. In addition, postovulatory follicles consistently showed positive staining. In both the large preovulatory and the postovulatory follicles, theca cells and granulosa cells were positively stained with the *lhcgrbb* antisense probe (see Fig. 3B, enlarged panels of antisense and sense probes). Next, we prepared a specific antibody for medaka Lhcgrbb protein. Affinity-purified antibodies recognized the medaka recombinant protein of the partial Lhcgrbb sequence used as antigen for the immunization of rats. While no immunoreactive materials were detected in the fish ovary extract by Western blotting using the antibodies, preliminary immunohistochemistry experiments with the antibodies reproducibly showed staining on the fish ovary sections. Based on the results of careful pilot experiments, the antibodies were judged to be suitable for immunohistochemical analysis of the ovary sections. We therefore detected Lhcgrbb protein immunohistochemically using anti-medaka Lhcgrbb antibody. Signals were found in the follicle layer of all sizes of growing follicles (Fig. 3C, left and middle panels). Positive staining was also detected in the oocyte cytoplasm of very small follicles, with a diameter less than 150 µm. In the large follicles, both granulosa and thecal cells were stained with the antibody (Fig. 3C, middle panel). The antibody previously treated with medaka recombinant Lhcgrbb did not give any positive signal (Fig. 3C, right panel).

### Specificity of medaka Fshra and Lhcgrbb

**Figure 4:**
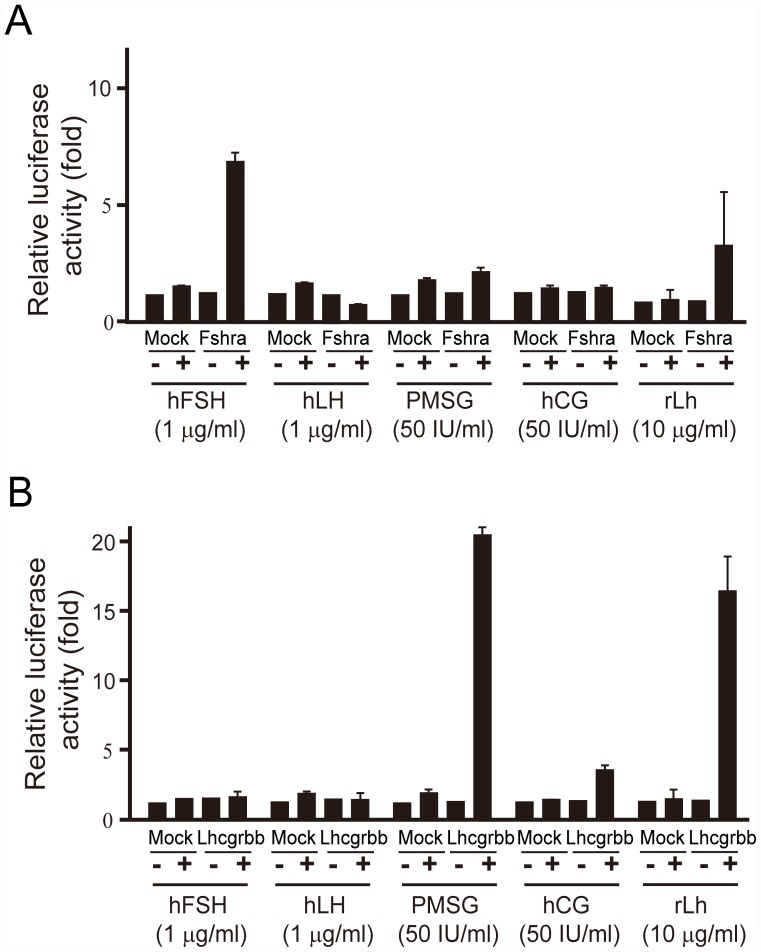
Specificity of medaka gonadotropin receptors. (A) HEK 293T cells expressing medaka Fshra were separately incubated with or without hFSH (1 µg/ml), hLH (1 µg/ml), PMSG (50 IU/ml), hCG (50 IU/ml), and rLh (10 µg/ml) for 24 h. Extracts of the treated cells were then prepared and assayed for luciferase activity. Relative enzyme activities are shown by setting the activity of the extract without hormone at 1. The results are presented as the mean ±S.E.M. (n = 6). (**B**) HEK293T cells expressing medaka Lhcgrbb were incubated with or without hormones, and the luciferase activity of the cell extracts was determined as in (**A**). The results are presented as the mean ±S.E.M. (n = 6).

The responsiveness of medaka gonadotropin receptors to various gonadotropins was examined using HEK 293T cells transfected with an expression vector, either pCMV-mFSHra or pCMV-mLHcgrbb. Medaka recombinant Fshra and Lhcgrbb were each expressed in the cells as a fusion protein with a FLAG tag at the C-terminus. The cells were simultaneously transfected with pGL4 Cre-luciferase vectors and pRL vectors. The treated cells were then incubated with hFSH, hLH, PMSG, hCG or medaka recombinant Lh (rLh) to determine luciferase activities. The luciferase activity was significantly increased only when the cells expressing medaka Fshra were treated with hFSH (Fig. 4A). Dose-dependency experiments indicated that the half-maximal effective concentration (EC_50_) of hFSH was 0.40±0.03 µg/ml (n = 3). In the cells expressing medaka recombinant Lhcgrbb, incubation with PMSG and medaka rLh resulted in increases in the luciferase activity (Fig. 4B). The EC_50_ values for PMSG, hCG, and medaka rLh were 16.5±0.9 IU/ml (n = 3), 26.0±3.1 IU/ml (n = 3), and 5.0±0.3 µg/ml (n = 3), respectively. In the present study, we failed to produce medaka recombinant Fsh in an active state. Therefore, analysis of the ligand/receptor interaction using Fsh and Fshra derived from the same species could not be performed.

### An ovulation system *in vitro* using *in vivo* non-Lh-surged preovulatory follicles in the presence of medaka rLh

**Figure 5:**
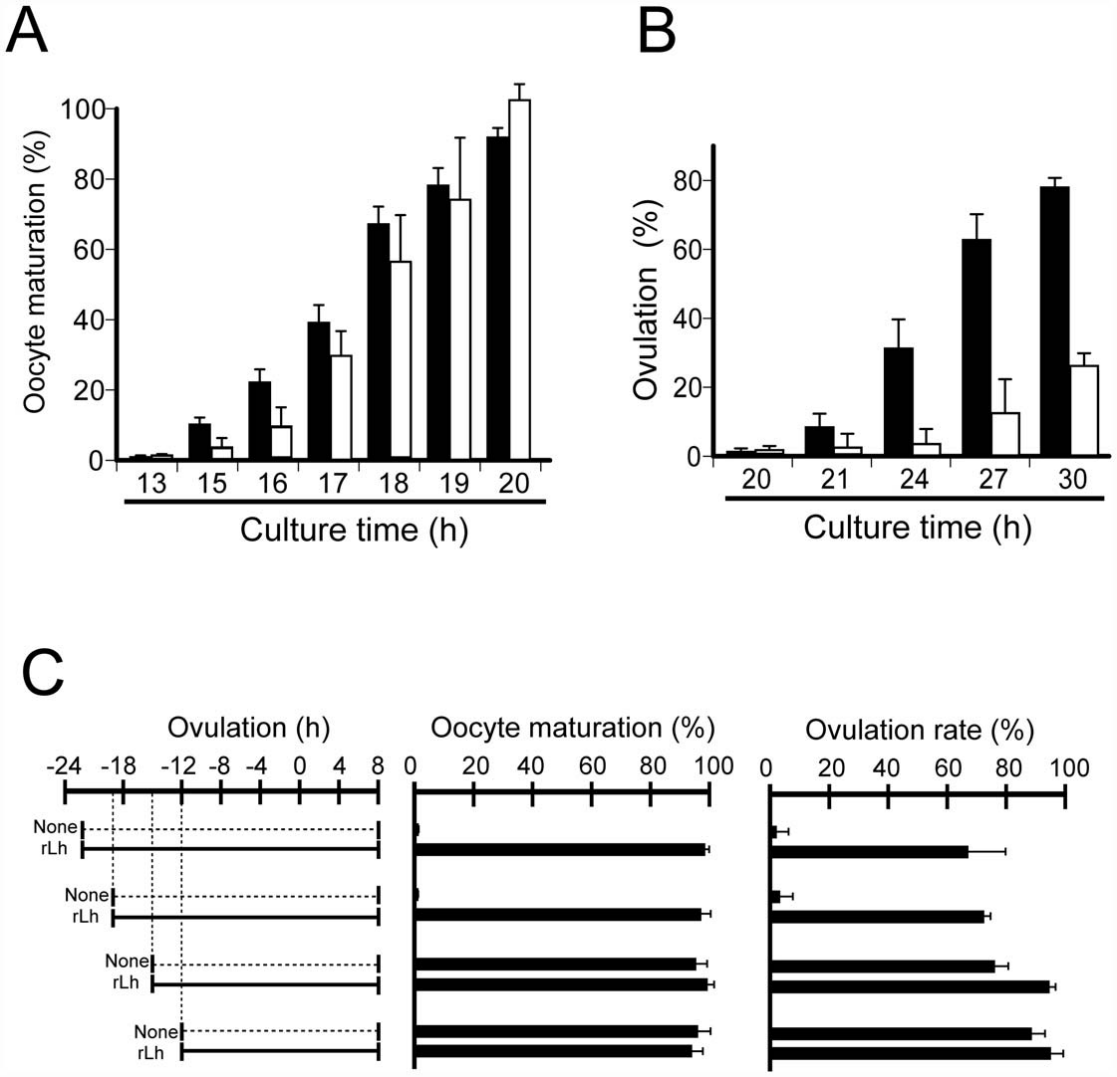
*In vitro* effect of rLh on oocyte maturation and ovulation of preovulatory follicles. (**A**) Preovulatory follicles were isolated 22 h before ovulation and cultured in medium containing either medaka rLh (10 µg/ml) (black bars) or PMSG (50 IU/ml) (white bars). The rates of oocyte maturation were determined every 2 h, and the results are presented as the mean ±S.E.M. (n = 5). (**B**) Preovulatory follicles were isolated and cultured in medium containing either medaka rLh (black bars) or PMSG (white bars) as in (**A**). The rates of follicle ovulation were determined every 2 h, and the results are presented as the mean ±S.E.M. (n = 5). (**C**) Preovulatory follicles were isolated 22, 19, 15, or 12 h before ovulation and cultured in medium containing rLh (10 µg/ml). At 8 h after the expected time of ovulation, oocytes showing GVBD or ovulated follicles were counted. The incubation conditions of isolated follicles (left) and the rates of oocyte maturation (middle) and ovulation in each condition (right) are shown. As controls, the follicles were incubated without rLh. A majority of follicles isolated 22 or 19 h before ovulation were dead at 8 h after ovulation when incubated without rLh, and therefore, the rates of oocyte maturation and ovulation were determined only with surviving follicles. The results are presented as the mean ±S.E.M. (n = 6).

Knowing that our current medaka rLh interacted with medaka Lhcgrbb in the experiment using HEK 293T cells, we examined the effect of medaka rLh on preovulatory follicles isolated from spawning medaka. When the postvitellogenic follicles were isolated from the ovaries 22 h before ovulation, they had not undergone the Lh surge *in vivo*. Cultured with rLh, the GVBD of the oocytes in the follicles was observed between 15 and 20 h after the start of incubation. Approximately 50% of the GVBD occurred at 17–18 h (Fig. 5A). For comparison, the effect of PMSG on the follicles *in vitro* was also examined. The GVBD in the oocytes of the follicles treated with PMSG occurred in a temporal pattern similar to that observed with the medaka rLh. Treatment of the follicles with these gonadotropins resulted in ovulation *in vitro* between 21 and 30 h of incubation (Fig. 5B). Approximately 50% of the follicles ovulated at approximately 25 h under these conditions. The rate of ovulation was much higher in rLh-treated follicles than in PMSG-treated follicles. The ovulation rate of rLh-treated follicles was comparable to that observed *in vivo*, while only 1/3 of the total follicles treated with PMSG ovulated successfully. Like the rLh-treated follicles, follicles incubated with PMSG, including the follicles that failed to ovulate, were all alive. The effects of hFSH and hCG on the preovulatory follicles were examined, but the gonadotropins had no effect on oocyte maturation or ovulation (data not shown). Inclusion of hFSH or hCG in the culture did not improve the rate of follicle survival.

To elucidate the *in vivo* timing of the Lh surge for the preovulatory follicles, the follicles isolated from the fish ovaries at different times during the 24-h spawning cycle were cultured in the presence or absence of rLh, and the ovulation rates were then determined (Fig. 5C). When the follicles isolated at 22 h before ovulation were used, only those cultured with medaka rLh were capable of ovulating. Similar results were obtained with follicles isolated 19 h before ovulation. More than 80% of the follicles isolated 15 or 12 h before ovulation ovulated even in the absence of medaka rLh. Interestingly, 85% of the follicles (105 of 124 follicles) isolated 22 h before ovulation and 76% of the follicles (99 of 131 follicles) isolated 19 h before ovulation died in the culture without rLh, while virtually all of the follicles incubated with the glycoprotein hormone survived. The results indicate the importance of exposure of the preovulatory follicle to Lh *in vivo* between 19 and 15 h before the time of ovulation.

We examined the quality of the oocytes ovulated from rLh-treated preovulatory follicles *in vitro* by fertilizing them *in vitro*. A total of 109 oocytes ovulated *in vitro* were collected for fertilization *in vitro*. Eighty-seven percent of the total oocytes were normally fertilized, and 90% of the fertilized oocytes developed into the blastula stage (stage 11). However, none of the embryos developed to the hatching stage. In preovulatory follicle culture experiments examining the effects of medaka rLh on oocyte maturation and ovulation using the culture medium containing the gonadotropin without purification, we obtained essentially the same results as those with the purified rLh sample (data not shown).

### Effect of medaka rLh on the expression of genes involved in follicle rupture during medaka ovulation

**Figure 6:**
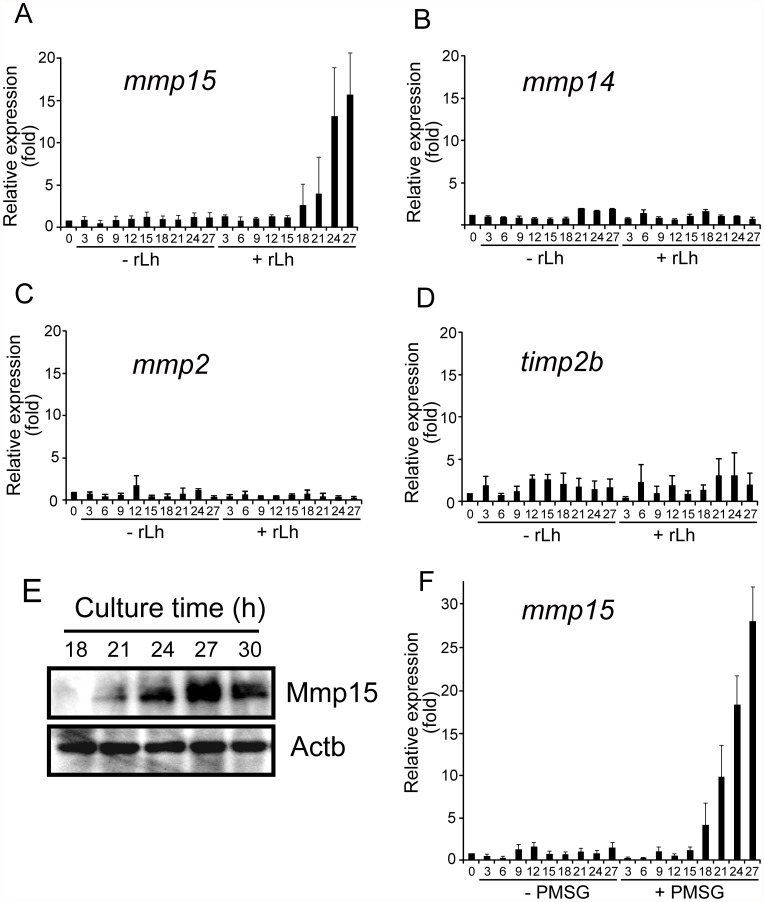
Induction of *mmp15* mRNA and protein expression in the preovulatory follicle by medaka rLh. Preovulatory follicles were isolated from spawning medaka ovaries 22 h before ovulation and incubated with mock conditioned medium (10 µg/ml) or medaka rLh (10 µg/ml). Follicles were collected every 3 h to extract total RNAs. Using the RNAs (2.5 µg each), real-time RT-PCRs were conducted for transcripts of *mmp15* (**A**), *mmp14* (**B**), *mmp2* (**C**), and *timp2b* (**D**). In the absence of rLh, the numbers of follicles with apparently abnormal morphology increased with incubation time from 0% (at the start of culture) to approximately 80% (at the end of culture), and therefore, the analyses were conducted using only surviving follicles with normal morphology. The expression levels were normalized to that of *actb* and expressed as the fold change compared to the levels of the −22 h follicle. The results are presented as the mean ±S.E.M. (n = 4). (**E**) Preovulatory follicles were isolated 22 h before ovulation and cultured in medium containing medaka rLh (10 µg/ml). The follicle layers of the follicles were collected at the indicated times and used for preparing follicle layer extracts. The extracts were analyzed by Western blotting using specific Mmp15 antibody. The proteins were loaded at 30 µg/lane. Medaka Actb was detected as a control. The reproducibility of the results was confirmed by conducting four separate experiments. The results of a representative experiment are presented. (**F**) Preovulatory follicles isolated from spawning medaka ovaries 22 h before ovulation were treated with or without PMSG (50 IU/ml). Collection of follicles, total RNA extraction, and real-time RT-PCR of *mmp15* were conducted as in (**A**). The expression levels were normalized to that of *actb* and expressed as the fold change compared to the levels of the −22 h follicle. The results are presented as the mean ±S.E.M. (n = 4).

Using the *in vitro* follicle culture system, we tested the ability of medaka rLh to induce mRNA expression of MT1-MMP (the official gene symbol is *mmp14*), MT2-MMP (*mmp15*), gelatinase A (*mmp2*) and TIMP-2b (*timp2b*), all of which are involved in follicle rupture during ovulation in the fish [15]. Large preovulatory follicles isolated from the spawning fish ovaries were incubated with or without rLh, and the levels of mRNA expression were determined by real-time RT-PCR. Follicular expression of *mmp15* mRNA was significantly induced by rLh (Fig. 6A), without an effect on the expression of *mmp14*, *mmp2*, or *timp2b* (Fig. 6, B, C, and D). Using the same *in vitro* culture, changes in Mmp15 protein level were examined by Western blot analysis of the rLh-treated preovulatory follicles. Mmp15 became detectable in the follicle layers of the follicles 21 h after the culture was started in the presence of medaka rLh (Fig. 6E). The protein level reached a maximum within 3–4 h after this.

Finally, we examined the effect of PMSG on the expression of the above four genes in the preovulatory follicles using our *in vitro* culture system. As in the case of medaka rLh, the expression of *mmp15* mRNA, but not *mmp14*, *mmp2*, or *timp2b* mRNA, was drastically induced by PMSG (Fig. 6F). The inducing effect of PMSG on *mmp15* mRNA expression in the follicles was temporally similar to that of medaka rLh; PMSG also induced *mmp15* mRNA expression at 18–27 h after the start of the gonadotropin treatment (Fig. 6A vs. 6F). *mmp15* mRNA expression was rather greater with PMSG exposure than with medaka rLh. These results indicate that, like medaka rLh, PMSG promotes *mmp15* mRNA expression in the preovulatory follicle.

## Discussion

In the present study, we obtained cDNA clones for *gtha*, *fshb*, *lhb*, *fshra* and *lhcgrbb* from medaka brains or ovaries. The amino acid sequences deduced from the nucleotide sequences of these clones showed homologies to the respective counterpart proteins from other species (Table 1). These data, together with the results of a functional characterization of recombinant proteins, demonstrate that the present clones represent medaka cDNA clones for *gtha*, *fshb*, *lhb*, *fshra* and *lhcgrbb* cDNA.

We showed that medaka gonadotropin subunit mRNAs (*gtha*, *fshb* and *lhb*) and proteins (Gtha, Fshb and Lhb) were expressed in the pituitary. This is in good agreement with the previous observation that all the mRNAs encoding the gonadotropin subunits are detectable in the fish pituitary [1], [28]. In addition to the pituitary, the fish testis, but not the ovary, expressed *fshb* mRNA at a detectable level. *fshb* mRNA is expressed in the ovaries of Southern catfish [29], gilthead seabream [30], zebrafish [31], and marbled eel [32]. In the zebrafish, *fshb* mRNA is also detectable in the testis [31]. Because the medaka testis extract contained no gonadotropin subunits, as determined by Western blotting using specific antibodies for the respective subunits, the biological meaning of the presence of *fshb* mRNA in this male reproductive organ is not clear at present. The expression levels of medaka pituitary *gtha*, *fshb* and *lhb* mRNAs periodically fluctuated in the 24-h spawning cycle, with the highest expression at ovulation but the lowest in the time between ovulations. In contrast, as shown by Western blotting, the subunit levels of the pituitary extract were generally constant during the spawning cycle, although virtually no subunits were detected 3 and 1 h before ovulation. This apparent inconsistency in the patterns of mRNA and protein levels in the fish pituitary during the 24-h spawning cycle may be due to their different turnover rates in the tissue. The fact that the gonadotropin subunits, especially the two Lh-constituting subunits Gtha and Lhb, exist in the pituitary of the spawning medaka between 23 and 7 h before ovulation is consistent with our idea that preovulatory follicles appeared to undergo an Lh surge between 19 and 15 h before ovulation. However, direct determination of Lh levels in the fish plasma is needed to support the validity of this idea. It is generally understood that the secretion of Lh is regulated by gonadotropin-releasing hormone (GnRH). The elucidation of the timing of an Lh surge leading to ovulation in medaka and the involvement of GnRH in the process await further studies.

By Western blot analysis, using specific antibodies for Gtha, Fshb, and Lhb, we consistently observed two polypeptides with similar molecular masses with each antibody. Because only a single mRNA species was detected for each of the corresponding gonadotropin subunits, it is reasonable to assume that a posttranslational modification may have caused the doublet of polypeptides. Differences in the carbohydrate attachment on the polypeptides might be responsible for the heterogeneity of the subunit polypeptides. Indeed, a multiplicity of gonadotropins generated by different extents of glycosylation has been reported for the gonadotropins of other teleost species [33]–[35].

Our current data strongly suggest that both granulosa cells and theca cells express Lhcgrbb in the preovulatory follicles of the fish ovary. On the other hand, *fshra* mRNA expression was observed in the follicle at stages up to late vitellogenesis but decreased to a negligible level when the follicle reached the postvitellogenic phase. This finding suggests that Fshra is absent in the large preovulatory follicles that are destined to ovulate. Such expression profiles of the gonadotropins in the medaka ovarian follicles are consistent with the previous results reported for other teleost species, supporting the hypothesis that Fsh/Fshra regulates the early phases of gametogenesis, such as vitellogenesis, whereas Lh/Lhcgrbb stimulates the final maturation stages, such as ovulation [1], [6], [8], [36].

Considering the observation that Lhcgrbb, but not Fshra, is expressed in the large preovulatory follicle, this gonadotropin receptor should be involved in the binding of the gonadotropin(s) that induce ovulation in the medaka. This hypothesis is further supported by the following results: i) using medaka Lhcgrbb-expressing HEK 293T cells, a highly specific interaction of medaka Lhcgrbb with rLh was demonstrated; ii) PMSG bound to medaka Lhcgrbb, but not to Fshra; and iii) both medaka rLh and PMSG induced *in vitro* ovulation of the preovulatory follicles. In this context, we should note the strictness of ligand-binding specificity of medaka Lhcgrbb because limited hormone-binding selectivity is a distinct feature of teleost gonadotropin bioactivity [31], [35], [37]–[45]. We found that treatment of the medaka Fshra-expressing HEK 293T cells with medaka rLh resulted in a slight increase in the luciferase activity. This finding might indicate a cross-activation of Fshra, that is, an interaction of Fshra with Lh in the medaka. To establish the strict specificities of medaka gonadotropin receptors, further studies are required. Nevertheless, the medaka gonadotropin receptors appear to be specific for their cognate gonadotropins compared with those from other teleost species. For example, zebrafish Fshra responds to goldfish pituitary extract and bovine FSH, while Lhcgrbb of the same fish can be activated by the pituitary extract, hCG, bovine FSH and bovine LH [31]. More recently, specificities of eel, trout, and tilapia gonadotropin receptors for various gonadotropins have been compared [46]. Generally, the results of that comparative study seem to support the broad specificity of fish gonadotropin receptors.

In the present study, we showed that rLh was a gonadotropin capable of inducing ovulation *in vitro* by postvitellogenic follicles in medaka. In addition, the follicles incubated with rLh synthesized Mmp15, an MMP indispensable for follicle rupture during ovulation in the fish [15]. These results indicate that Lh is involved in fish ovulation. Our data also indicate that the preovulatory follicles that are destined to ovulate undergo an Lh surge between 19 and 15 h before ovulation *in vivo*. It should be noted that we failed to produce medaka recombinant Fsh in the present study. This did not allow us to test the potential binding of medaka Fsh to medaka Lhcgrbb. Therefore, we cannot exclude completely the possibility that Fsh plays a role in medaka ovulation.

It is worthwhile to consider the relevance and usefulness of the *in vitro* rLh-supplemented follicle culture system reported in this study. To date, many *in vitro* methods using ovary fragments and ovarian follicles have been elaborated for various teleost species. They include zebrafish [47]–[49], Atlantic croaker [50], [51], rainbow trout [52], [53], brook trout [54], goldfish [55], [56], sea lamprey [57], coho salmon [58], European sea bass [59], and killifish [60]. Although these experimental models generally are good systems for studying oocyte maturation of the respective teleost species, mature, healthy and intact oocytes cannot come off the follicle or ovarian fragments even when they have been primed by gonadotropins *in vivo*. To our knowledge, the current rLh-supplemented culture method using medaka preovulatory follicles *in vitro* is the only experimental system that allows the follicles to undergo oocyte maturation as well as ovulation at rates as efficient as *in vivo*. PMSG has an Lh-like activity on medaka preovulatory follicles [61]. Our present experiments using HEK 293T cells expressing medaka Lhcgrbb confirmed the binding of PMSG to Lhcgrbb, indicating that PMSG can mimic the action of Lh in the preovulatory follicles. GVBD and the ovulation-inducing effect of PMSG on the follicles were confirmed with the *in vitro* follicle culture system. Further, we showed that, like rLh, PMSG drastically induced *mmp15* mRNA expression in the preovulatory follicle. However, the effect of PMSG on follicle ovulation was not as efficient as that of rLh. At present, we have no clear explanation for the difference in the efficiency between rLh and PMSG. We only speculate that PMSG may not be able to induce other ovulation-related genes/proteins as effectively as medaka rLh.

**Figure 7:**
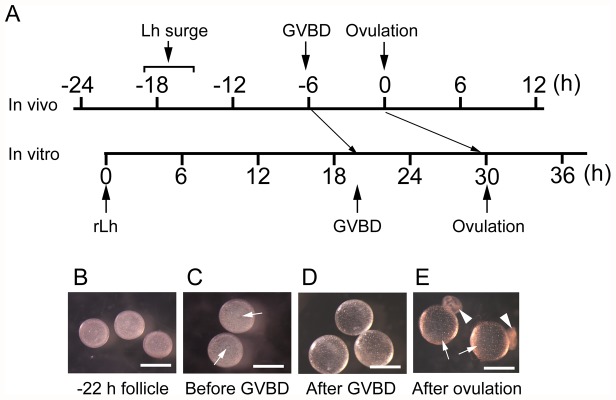
Comparison of the timetable of medaka oocyte maturation and ovulation *in vivo* and *in vitro*. (**A**) The expected times of the Lh surge, GVBD and ovulation *in vivo* are shown (upper line). The timetable of GVBD and ovulation observed with the follicles incubated in the presence of medaka rLh is shown (lower line). (**B**) Follicles that had not yet undergone the LH surge *in vivo*, isolated from ovaries 22 h before ovulation. (**C**) Follicles incubated with rLh for 12 h (before GVBD). The arrows indicate germinal vesicles. (**D**) Follicles incubated with rLh for 20 h (after GVBD). (**E**) Successfully ovulated follicle *in vitro*. The arrow and arrowhead indicate the oocyte and the follicle layer, respectively. Bars represent 1 mm.

A schedule of GVBD and ovulation in the follicles cultured with rLh *in vitro* is depicted in Fig. 7, in which the schedules of the two processes under physiological conditions are also shown for comparison. Preovulatory follicles isolated 22 h before the expected time of ovulation undergo GVBD and ovulation at 20 h and 30 h, respectively, after the start of incubation in the presence of rLh. These two biological processes go slowly and require a long time for completion. It takes 5 h and 9 h for completion of GVBD and ovulation, respectively, in our *in vitro* system. This is a notable difference compared with the *in vivo* situation; GVBD occurs approximately 6 h before ovulation and the subsequent ovulation within 0.5 h before the onset of light *in vivo* [61]–[63], and both are completed in a short period of time. Therefore, GVBD and ovulation are delayed 4 and 8 h, respectively, under our culture conditions *in vitro*. The considerable delay of these processes might be explained as follows. In the follicles separated from the ovary 22 h before ovulation for *in vitro* culture, various substances, including nutritional materials and other necessary factors, may no longer be provided through the blood system, and a shortage of such substances may cause the substantially reduced activity of the follicles. A similar explanation may account for the limited developmental ability of the embryos when the oocytes derived from the *in vitro* rLh-supplemented ovulation were fertilized; the embryos developed to the blastula stage but not to the hatching stage. Nevertheless, although oocyte maturation and ovulation are delayed, the *in vitro* follicle culture model elaborated in the present study is obviously a useful experimental system for oocyte maturation and ovulation studies.

Expression of various genes and proteins is induced at or around the time of ovulation in teleosts. They include trout ovulatory protein-2 mRNA [64], [65]; amago salmon 20β-hydroxysteroid dehydrogenase activity [66]; medaka *star* and *cyp17a1* mRNAs [6]; rainbow trout serine protease 23, *adam22*, *cscl14*, *fgf2*, and *ace2* mRNAs [67]; medaka prostaglandin E2 receptor mRNA [68]; and channel catfish *cyp11a*, *cyp17a1*, *cyp19*, and *hsd3b* mRNAs [44]. Among the genes and proteins described above, the channel catfish *cyp11a*, *cyp17a1*, *cyp19*, and *hsd3b* mRNAs are up-regulated by Lh [44]. The present study demonstrates that medaka Mmp15 is undoubtedly one of the Lh-induced proteins. In contrast, a large number of ovulation-related genes induced by the LH surge have been identified in mammals [17]. Our current *in vitro* culture method using preovulatory follicles should serve as a powerful experimental system for exploring the endocrine mechanism underlying follicle rupture during ovulation, including the induction of ovulation-related genes.

Oocyte maturation is regulated by the non-genomic action of maturation-inducing hormone (MIH), while ovulation is regulated by genomic mechanisms [69], [70]. Nagahama and Yamashita reported that gonadotropin induces rapid expression of nuclear progesterone receptor (nPR) mRNA in granulosa cells [6]. Because 17α,20β-dihydroxy-4-pregnen-3-one (DHP), which is the naturally occurring MIH for many teleost fishes [11], is a ligand for nPR [71] and because DHP levels in the plasma are strikingly elevated in mature and ovulated females in response to Lh [6], the activation of nPR by DHP binding could be an initial step in the transcription of ovulation-related genes. These considerations lead us to speculate that nPR is involved in the substantial induction of *mmp15*/Mmp15 at ovulation in the medaka. Studies are now under way in our laboratory to establish the role of nPR in the ovarian expression of *mmp15* using our *in vitro* follicle ovulation method.

In conclusion, we carried out expression and characterization studies of medaka gonadotropins. We showed that Lh/Lhcgrbb is critically involved in the induction of oocyte maturation and ovulation, although the involvement of Fsh/Lhcgrbb in these processes cannot be excluded completely. Using rLh produced in this study, an *in vitro* follicle ovulation method was developed. To the best of our knowledge, this method is the only experimental system at present that allows us to follow the whole process of oocyte maturation and subsequent ovulation in teleosts. The usefulness of the method was demonstrated by examining the mRNA expression of three MMPs and *timp2b*, which are involved in medaka ovulation [15], in the follicles using this culture method. Information obtained from the present study will help researchers conduct future studies to clarify the molecular mechanism of ovulation from an endocrinological perspective.

## Materials and Methods

### Medaka culture and tissue preparation

Medaka fish were purchased from a local dealer. The fish were acclimated to conditions of 27°C and a lighting regime of 14 h light and 10 h dark. After becoming acclimated to these conditions, females usually spawn daily within 0.5 h of the onset of light [62], [63]. Medium-sized follicles (0.5–1.0 mm, vitellogenic phase, stage VII-VIII) and large follicles (0.8–1.2 mm, postvitellogenic phase, stage IX-X) were isolated from the ovaries. In the fish, large follicles undergo the Lh surge, the breakdown of the germinal vesicle (GVBD), and ovulation at approximately 21, 6, and 0 h, respectively, before the start of the light period [62], [63]. Ovarian follicles were staged as described previously [72]. The start of the light period was designated as ovulation 0 h in this study. Pituitaries were isolated from adult female medaka at the indicated times. Other tissues were obtained 3 h after ovulation. All the animal treatments were carried out according to the guidelines for animal experiments at Hokkaido University and were approved by the Committee of the Experimental Plants and Animals, Hokkaido University (Permit Number: 19(11) and 23(30)).

### Follicle culture and *in vitro* ovulation

Large follicles were isolated from ovaries 22, 19, 15, or 12 h before ovulation and incubated at 26°C in a 35-mm-diameter culture dish containing 90% M199 medium (4 ml per dish) (Dainippon-Sumitomo Seiyaku, Osaka, Japan) and 50 µM gentamycin (pH 7.4). Follicles were isolated from three to five fish ovaries, each of which provided 15–25 follicles. The follicles were then pooled and used for experiments. Approximately 20 follicles were used per incubation. Hormones used for incubation of follicles *in vitro* were human follicle-stimulating hormone (hFSH) (Sigma-Aldrich, St. Louis, MO), pregnant mare’s serum gonadotropin (PMSG) (Sigma-Aldrich), human luteinizing hormone (hLH) (Sigma-Aldrich), and human chorionic gonadotropin (hCG) (Sigma-Aldrich). Medaka recombinant Lh (rLh) was prepared in this study. The numbers of follicles successfully ovulated were counted at 8 h after the expected time of ovulation, and the ovulation rates were calculated. The viability of follicles at the end of each culture period was evaluated by staining with 0.08% trypan blue. *In vitro* fertilization was performed as previously described [14]. The developmental stage of the embryos was determined as previously described [73].

### RNA isolation, RT-PCR, and real-time PCR

Total RNA was isolated using Isogen (Nippon Gene, Tokyo, Japan) according to the manufacturer’s instructions. The RNA (2.5 µg) was reverse-transcribed using SuperScript® III Reverse Transcriptase (Invitrogen, Carlsbad, CA) with Oligo (dT)_20_ Primer (Invitrogen) according to manufacturer’s instructions. The resultant cDNAs were PCR-amplified in a volume of 10 µl that consisted of RT reaction, 1× Ex Taq buffer, 0.2 mM of each dNTP, 0.5 µM of each primer, and 0.25 U *TaKaRa Ex Taq* ^®^ Hot Start Version (Takara, Tokyo, Japan). The PCR conditions were hot start at 94°C for 3 min, followed by denaturation at 94°C for 30 sec, annealing at 60°C for 30 sec, and extension at 72°C for 30 sec for 20 or 30 cycles. Real-time PCR was performed using an Applied Biosystems 7300 Real-Time PCR System (Life technologies Inc., Rockville, MD). The PCR mixture preparation, PCR, and data analysis were carried out according to the previously reported protocol [68], [74]. To normalize the transcript levels of gonadotropin subunits (pituitary), gonadotropin receptors, metalloproteinases and their inhibitor (ovarian follicles), we tested the housekeeping genes *cytoplasmic actin* (*actb*), *18S rRNA* (*rn18s1*) and *ribosomal protein L7* (*rpl7*). The most stably expressed gene in the tissues was *actb*, and therefore *actb* mRNA expression was used for normalization. The primer pairs used are shown in Table S1.

### cDNA cloning of gonadotropin subunits and receptors

To obtain cDNA fragments for gonadotropin subunits and receptors, RT-PCR was conducted using KOD-Plus-Neo DNA polymerase (Toyobo, Tokyo, Japan) with a brain cDNA for medaka Gth α-subunit (official symbols: *gtha* for the gene and Gtha for the protein), Fsh β-subunit (*fshb* for the gene and Fshb for the protein) and Lh β-subunit (*lhb* for the gene and Lhb for the protein), or with ovary cDNAs for Lh receptor (*lhcgrbb* for the gene and Lhcgrbb for the protein) and Fsh receptor (*fshar* for the gene and Fshra for the protein). The PCR conditions were 94°C for 2 min, followed by denaturation at 94°C for 30 sec, annealing at 60°C for 30 sec, and extension at 68°C for 1 min (for *gtha*, *fshb*, and *lhb*) or 5 min (for *fshra* and *lhcgrbb*) for 30 cycles. The primers used are listed in Table S1. Amplified products were gel-purified, ligated into pBluescript II KS (+) (Stratagene, La Jolla, CA), and the nucleotide sequences of the inserted fragments were confirmed by sequencing. As a result, cDNA clones for *gtha* (487 bp), *fshb* (471 bp), *lhb* (529 bp), *fshra* (2130 bp) and *lhcgrbb* (2140 bp) were obtained.

### Northern blot analysis

Total RNAs (60 µg) were separated on a 1.2% agarose gel containing 6% formaldehyde and transferred to a Hybond-N^+^ membrane (GE Healthcare, Buckinghamshire, England). The cDNA fragments for *gtha* (nucleotides 1–487, AB541980), *fshb* (nucleotides 152–520, AB541981), *lhb* (nucleotides 3–531, AB541982), *fshra* (nucleotides 168–647, AB526237), and *lhcgrbb* (nucleotides 27–530, AB526238) were used as probes. The membrane was hybridized with a [α-^32^P] dCTP-labeled probe at 65°C for 18 h in 5% SDS, 1% BSA, 0.1 mM EDTA, and 0.5 M sodium phosphate (pH 7.2). After hybridization, the membrane was washed twice in 2× saline sodium citrate (SSC) (0.15 M NaCl, 15 mM sodium citrate) containing 0.1% SDS at 65°C for 30 min and then washed twice in 0.1× SSC containing 0.1% SDS at 65°C for 30 min. As a control, medaka cytoplasmic actin (*actb*) mRNA was detected with a [α-^32^P] dCTP-labeled 312-bp *actb* cDNA fragment [68], [75]. The membrane was exposed to X-ray film (Kodak, Tokyo, Japan), which was then developed.

### *In situ* hybridization

A cDNA fragment for *fshra* (480 bp), which corresponded to the nucleotide sequence 168–647 (AB526237), or *lhcgrbb* (252 bp), which corresponded to the nucleotide sequence 1341–1592 (AB526238), inserted into pBluescript was used as the template for *in vitro* transcription. Both antisense and sense digoxigenin (DIG)-labeled riboprobes were generated with T3 or T7 RNA polymerase and a DIG RNA Labeling Mix (Roche Diagnostics, Manheim, Germany). *In situ* hybridization was performed as previously described [15]. Briefly, frozen sections (12 µm) of the ovaries isolated 1 h after ovulation were fixed, acetylated, and hybridized at 50°C for 18 h in a solution containing 50% formamide, 0.5 M NaCl, 10 mM Tris-HCl (pH 8.0), 1 mM EDTA (pH 8.0), 10% dextran sulfate (Wako, Osaka, Japan), 1× Denhardt’s solution (Wako), 0.25% SDS, and 0.2 mg/ml yeast transfer RNA. After hybridization, the sections were washed, and the signals were detected. For *in situ* hybridization of *lhcgrbb*, paraffin sections (5 µm) were prepared using ovaries isolated 1 h after ovulation and fixed in Bouin’s solution. The specimens were deparaffinized and permeabilized in PBS containing 0.3% Triton-X 100 for 5 min at room temperature. After washing in PBS three times, they were incubated in PBS containing 0.2 M HCl for 5 min at room temperature. They were washed in PBS three times and incubated in PBS containing 50 µg/ml Proteinase K (Roche, Diagnostics) for 10 min at 37°C. After washing again in PBS three times, they were fixed in PBS containing 4% paraformaldehyde (Wako) for 15 min at RT. They were washed in PBS three times and acetylated in 0.1 M triethanolamine (pH 8.0) containing 0.25% acetic anhydride for 10 min at RT. After being washed in PBS three times, they were prehybridized at RT for 1 h in 50% formamide, 1× Denhardt’s solution, and 0.2 mg/ml yeast transfer RNA. Then, they were hybridized at 50°C for 16 h in the same buffer used for prehybridization. After hybridization, they were washed in 5× SSC for 5 min at 50°C, 2× SSC containing 50% formamide for 30 min at 50°C, 2× SSC for 20 min at 50°C, and 0.2× SSC for 20 min at 50°C. Positive signals were detected as previously described [15].

### Primary culture of medaka granulosa cells

Granulosa cells were isolated from spawning female fish ovaries as previously described [27]. Briefly, large follicles were isolated from ovaries (at least 3 ovaries) 3 h before ovulation, punctured to remove the egg yolk, and centrifuged at 900×*g* for 5 min. The precipitated materials were suspended in phosphate-buffered saline (PBS) containing 0.25% trypsin and 1 mM EDTA and kept for 60 min at room temperature with gentle rotation. The trypsin-treated samples were washed with PBS and passed through a 100 µm nylon filter (BD Biosciences, Bedford, MA). The resulting filtrates were cultured at 26°C in 90% M199 medium, pH 7.4, containing 50 µM gentamycin and 10% FBS (Wako). After 48 h of culture, cells were collected and stored at −80°C until they were used.

### Preparation of pCMV vector constructs

Coding regions for *fshra* (nucleotides 171–2231, AB526237) and *lhcgrbb* (nucleotides 27–2090, AB526238) were amplified by PCR with KOD-Plus-Neo DNA polymerase using the cDNA fragments inserted into pBluescript II KS (+) as templates. The primers used are listed in Table S1. The PCR products were digested with *Eco*RI and *Hind*III and ligated into the pCMV vector tag4 (Stratagene), which had been digested with the same enzymes. The sequences of the resulting vectors, named pCMV-mFSHra and pCMV-mLHcgrbb, respectively, were confirmed by sequencing.

### Functional analysis of recombinant Fshra and Lhcgrbb

HEK 293T cells were cultured in DMEM (Wako) containing 10% FBS and 1×PSG (Invitrogen). Either pCMV-mFSHra or pCMV-mLHcgrbb and the pGL4 Cre-luciferase vector (Promega, Madison, WI), which contained the cAMP-response element (CRE) and luciferase in the 5’ upstream region and 3’ downstream region of its promoter, respectively, along with the pRL vector (Promega), which was used for internal normalization of luciferase expression measurements, were simultaneously transfected into cells using Lipofectamine 2000 (Invitrogen) in Opti-MEM I medium (Invitrogen). Beginning 24 h after the start of culture, the cells were incubated for an additional 24 h in medium containing hFSH, PMSG, hLH, hCG, or medaka rLh. The luciferase activities of the treated cells were measured using the Dual-Luciferase Reporter Assay System (Promega) according to the manufacturer’s instructions.

### Preparation of medaka rLh

A fusion cDNA containing the *lhb* and *gtha* sequences in that order was prepared. Specifically, coding regions of the entire sequence of *lhb* and of the *gtha* sequence without its putative signal peptide were separately amplified by PCR from pBluescript II vectors containing either the *lhb* or the *gtha* sequence using KOD-Plus-Neo DNA polymerase. The primer sets used were Lh pEB SS and Lhβ+Gthα AS for *lhb* amplification and LHβ+GTHα SS and Gthα pET AS for *gtha* amplification (Table S1). The respective amplified products were gel-purified, mixed, and used as templates for further PCR with Lh pEB SS and Gthα pET AS. The product was digested by *Kpn* I and *Xho*I and then ligated into the pEB Multi-Neo vector (Wako) previously digested with the same restriction enzymes. Using the resulting vector, it was possible to establish stable cell lines without integration into the host genome. The cDNA sequence of the vector, named pEB-LH, was confirmed by sequencing.

Chinese hamster ovary (CHO) K-1 cells were cultured at 37°C in F-12 medium (Wako) supplemented with 5% FBS, 1× penicillin-streptomycin-amphotericin B suspension (Wako), and 2 mM L-glutamine solution (Wako). The pEB-LH vector was transfected into the cells using Lipofectamine 2000 and Opti-MEM medium according to the manufacturer’s instructions. Forty-eight hours after starting the transfection, the medium was changed to F-12 medium containing 0.5 mg/ml geneticin sulfate (Invitrogen) and the supplements described above. The cells were cultured for 14 more days with medium changes every 2 days. For the final 7 days, F-12 medium without FBS and geneticin sulfate was used. The spent medium (approximately 1,000 ml) was collected and concentrated 20-fold. The concentrated medium was dialyzed against buffer A (50 mM Tris-HCl (pH 8) and 0.5 M NaCl) and applied to concanavalin A agarose (GE Healthcare) previously equilibrated with buffer A. After washing with buffer A containing 10 mM methyl-α-mannopyranoside (Sigma), the bound proteins were eluted with the same buffer containing 0.2 M methyl-α-mannopyranoside. The eluted fractions were pooled, dialyzed against buffer B (50 mM Tris-HCl (pH 8)), and applied to a Resource Q column attached to an AKTA purifier (GE Healthcare). The column was operated at a flow rate of 1 ml/min. After washing with buffer B, bound materials were eluted with buffer B containing 0.1 M NaCl. The eluted sample (total volume, 10 ml; protein concentration, 0.13 mg/ml) was stored at −80°C until it was used.

### Preparation of recombinant proteins and production of their specific antibodies

Medaka recombinant Gtha, Fshb, Lhb, and Lhcgrbb were produced using an *E. coli* expression system. The coding regions of the gonadotropin subunit polypeptides without predicted signal sequences and a partial sequence corresponding to amino acid residues 27–362 of Lhcgrbb were amplified by PCR with KOD-Plus-Neo DNA polymerase using the respective cDNA fragments inserted into pBluescript II KS (+). The primers used are listed in Table S1. The PCR products were digested by *Eco*RI and *Xho*I for *gtha* and *Eco*RI and *Hin*dIII for *fshb*, *lhb*, and *lhcgrbb*. The digested products were then ligated into the pET30a vector (Novagen, Madison, WI) previously digested with the same restriction enzymes. The resulting vectors were confirmed by nucleotide sequencing and transformed into the *E. coli* Rosetta strain (Novagen). Recombinant protein expression and purification on a Ni^2+^-Sepharose column were performed as previously described [76]. Using the purified recombinant protein, mice (for Gtha, Fshb, and Lhb) or rats (for Lhcgrbb) were immunized to produce specific antisera. For the primary immunization, 80 µg (for mice) or 300 µg (for rats) of each of the recombinant proteins was mixed into a water-in-oil emulsion using an equal volume of Freund’s complete adjuvant (Wako). Boosting was started 2 weeks later by injecting at 2-week intervals 40 µg (for mice) or 300 µg (for rats) of each of the recombinant proteins in Freund’s incomplete adjuvant (Wako). Boosting injections were conducted four times. Sera were collected 2 weeks after the final injection. Specific antibodies were affinity-purified using membranes on which pure antigens were blotted. Anti-medaka Mmp15 antibody [15] and anti-medaka Actb antibody [16] were prepared as previously described.

### Tissue extract preparation and Western blot analysis

Tissues were homogenized in PBS containing 1 mM EDTA and a protease inhibitor mixture (Wako) and centrifuged at 15,000×*g* for 10 min to separate the supernatant and insoluble fractions. The resulting supernatant was used directly for Western blot analysis to detect gonadotropin subunit proteins. The precipitated materials were boiled in PBS containing 1% SDS for 20 min and then centrifuged at 15,000×*g* for 10 min. The resulting supernatant was used for Western blot analysis to detect Mmp15 protein. Extracts of follicle layers were prepared as previously described [15]. The protein concentrations were determined using a Pierce BCA Protein Assay Reagent kit (Thermo Fischer Scientific, San Jose, CA). Sodium dodecyl sulfate–polyacrylamide gel electrophoresis (SDS-PAGE) and immunoblotting with Immobilon PVDF membranes were performed following standard procedures, except secondary antibodies were reacted in 1×IP/WB Optima E Dilution Reagent (Santa Cruz Biotechnology, Inc., Santa Cruz, CA). Signals were detected using an Immobilon Western kit (Millipore, Bedford, MA) according to the protocol provided by the manufacturer.

### Immunohistochemistry

Paraffin sections (5 µm) were used for immunohistochemistry. Ovaries were isolated from spawning medaka 1 h after ovulation and fixed in PBS containing 4% paraformaldehyde (Wako). The sections were deparaffinized and incubated in PBS containing 3% H_2_O_2_ for 10 min at RT. After three washes in PBS, they were incubated in Block Ace (Dainippon-Sumitomo Seiyaku) for 1 h at RT. The sections were then incubated with anti-medaka Lhcgrbb antibody for 1 h at RT and washed in PBS three times. After washing, they were reacted with anti-rat IgG antibody (GE healthcare) for 1 h at RT. After three washes in PBS, the sections were stained using an AEC kit (Vector Laboratories, Burlingame, CA) according to the manufacturer’s instructions. For the negative control, the primary antibody was preincubated with antigen (20 µg) for 16 h at 4°C, and the treated antibody was then used for immunohistochemistry.

### Statistical analysis

All of the experiments were repeated at 3 to 8 times, except the Northern blot analyses, for which two independent experiments were performed. Error bars indicate the standard error of the mean (S.E.M.) obtained from 3 to 8 independent experiments. Statistical analysis was performed by Student’s *t-*test. A *P* value of less than 0.05 was considered statistically significant.

## Supporting Information

Table S1 **Primers used in this study.**
(DOC)
