# Supplementary material for: Characterization of Luteinizing Hormone and Luteinizing Hormone Receptor and Their Indispensable Role in the Ovulatory Process of the Medaka
Source: PLoS One. 2013 Jan 23;8(1):e54482. doi: 10.1371/journal.pone.0054482 (PMC3553140; doi:10.1371/journal.pone.0054482)
Supplement: Table S1 — Primers used in this study. (DOC) [file pone.0054482.s001.doc]

**Table S1.** Primers used in this study.

Primer name gene Sequence Accession No.

Cloning, Northern blotting

Gthα SS *gtha* 5′-ATAAAGGAGTCCTGCAGAAC-3′ AB541980

Gthα AS *gtha* 5′-AGAATCCAACTCTATTCCAA-3′

Fshβ SS *fshb* 5′-CAGTGACGCTGGAGATCTACA-3′ AB541981

Fshβ AS *fshb* 5′-CACAGTTTCTTTATTTCAGT-3′

Lhβ SS *lhb* 5′-AGCACACAGCCTGCAGATA-3′ AB541982

Lhβ AS *lhb* 5′-CAGTTTTAATTGAATAAGGC-3′

Cloning

Fshr SS-F *fshra* 5′-TGCTGTTAGGGACAAAAGCTGACT-3′ AB526237

Fshr AS-F *fshra* 5′-CACTAAACTGTGTACTGGTGGATA-3′

Lhr SS-F *lhcgrbb* 5′-GGATGAGCTGTGGCCCGCCGGCGC-3′ AB526238

Lhr AS-F *lhcgrbb* 5′-TAACTCTCCATTTTGATCTAGCTT-3′

Northern blotting

Fshβ SS-2 *fshb* 5′-CCAGCTTTTCCTGTCATCCC-3′ AB541981

Lhr SS *lhcgrbb* 5′-ATGGCTCCCCGGGTGGTCTGG-3′ AB526238

Lhr AS *lhcgrbb* 5′-GGAGTTTGCAGGAATCACGTC-3′

MT2-MMP SS *mmp15* 5′-CTCAGTGACGATGGAGC-3′ AB072928

MT2-MMP AS *mmp15* 5′-GCGTGTTGATGATGGCGTAG-3′

Northern blotting, RT-PCR, in situ hybridization

Fshr SS *fshra* 5′-AGTATGGTGGTAATGATACAG-3′ AB526237

Fshr AS *fshra* 5′-AATCCTCAAACCCGTGTTGGT-3′

β-actin SS *actb* 5′-CAGACACGTATTTGCCTCT-3′ D89627

β-actin AS *actb* 5′-CAAGTCGGAACACATGTGCA-3′

in situ hybridization

Lhr SS-2 *lhcgrbb* 5′-GCCGGCTTTTTGACAGTGTTT-3′ AB526238

Lhr AS-2 *lhcgrbb* 5′-CTGAGAGCTCAGGGTGTCGATG-3′

RT-PCR

GelB SS *mmp9* 5′- CAAAACAGATCCTAAACCAACTGT-3′ AB033755

GelB AS *mmp9* 5′-ATTTTAGGAGATCATATTTCACGT-3′

Coll-1 SS *col1A1* 5′-GAGAAATCTGGACTTGAA-3′ AB280535

Coll-1 AS *col1A1* 5′-GTACAGAGCAACCGAGTT-3′

Coll-4 SS *col4A1* 5′-ACCGGTGTTCTGGCTTTAC-3′ AB525196

Coll-4 AS *col4A1* 5′-TTGGGGCGTGAAATCAGTG-3′

Table S1 (continued)

Real-time PCR

Gthα real-t SS *gtha* 5′-CTGCAGCACCTGCTATTATCA -3′ AB541980

Gthα real-t AS *gtha* 5′-GGTATTGCCAAATACGAGCAG-3′

Fshβ real-t SS *fshb* 5′-TGGAGATCTACAGGCGTCGGTAC-3′ AB541981

Fshβ real-t AS *fshb* 5′-AGCTCTCCACAGGGATGCTG-3′

Lhβ real-t SS *lhb* 5′-TGCCTTACCAAGGACCCCTTGATG-3′ AB541982

Lhβ real-t AS *lhb* 5′-AGGGTATGTGACTGACGGATCCAC-3′

Fshr real-t SS *fshra* 5′-CAAGGCACCTGACTTCCATC-3′ AB526237

Fshr real-t AS *fshra* 5′-AAATCCTCAAACCCGTGTTG-3′

Lhr real-t SS *lhcgrbb* 5′-TCCTCTCCCTCCACAGTCTG-3′ AB526238

Lhr real-t AS *lhcgrbb* 5′-GATGGTCAAAAACTCCAGCC-3′

GelA real-t SS *mmp2* 5′- GCGACGACGGCTTTTTGTGG-3′ AB033754

GelA real-t AS *mmp2* 5′- CATCTCCATTCCCTCCCAG-3′

MT1 real-t SS *mmp14* 5′-CGGAGGGTTTCCACGGCGAC-3′ AB185847

MT1 real-t AS *mmp14* 5′-CCAATCGTCCACGGCTCAGC-3′

MT2 real-t SS *mmp15* 5′- ACGGCTCCCTCCAGTTTTAC -3′ AB072928

MT2 real-t AS *mmp15* 5′- GGTGTTGTTCCTGCGTCTTC-3′

TIMP2b real-t SS *timp2b* 5′-TGGAGACTGATGGGACGATG-3′ AB193468

TIMP2b real-t AS *timp2b* 5′-GCACGCAGGGAATGGAAGTG-3′

β-actin real-t SS *actb* 5′-TGACGGAGCGTGGCTACTC-3′ D89627

β-actin real-t AS *actb* 5′-TCCTTGATGTCACGGACAATTT-3′

Recombinant protein preparation

Gthα pET SS *gtha* 5′-CCGGAATTCGAATGCCGGCTGGAG-3′ AB541980

Gthα pET AS *gtha* 5′-CCGCTCGAGTCACATCTTGTG -3′

Fshβ pET SS *fshb* 5′-CCGGAATTCCAGGTCTCCAGCTTT-3′ AB541981

Fshβ pET AS *fshb* 5′-CCCAAGCTTTTAACAGCTCGGCAT-3′

Lhβ pET SS *lhb* 5′-CCGGAATTCCCTTACTGCCAGCCA-3′ AB541982

Lhβ pET AS *lhb* 5′-CCGAAGCTTCTAATAATAAAAAGA-3′

Lhr pET SS *lhcgrbb* 5′-CCGGAATTCCGCTCCTGCCAGGCG-3′ AB526238

Lhr pET AS *lhcgrbb* 5′-CCCAAGCTTTTACAGCAGGTCCTCGCA-3′

Lh pEB SS *lhb* 5′-CGGGGTACCATGATTTCCCGG-3′                  AB541982

Lhβ+Gthα SS 5′- GATTCTTTTTATTATGACACCTACTCCAAT-3′

Lhβ+Gthα AS 5′- ATTGGAGTAGGTGTCATAATAAAAAGAATC-3′

Fshr pCMV SS *fshra* 5′-CCGGAATTCATGGTGGTAATGATA-3′ AB526237

Fshr pCMV AS *fshra* 5′-CCCAAGCTTGCACTCTTGTTTCCC-3′

Table S1 (continued)

Lhr pCMV SS *lhcgrbb* 5′-CCGGAATTCATGGCTCCCCGGGTG-3′ AB526238

Lhr pCMV AS *lhcgrbb* 5′-CCCAAGCTTCGTGGCTCCTTCACT-3′
